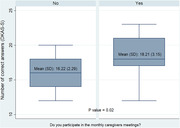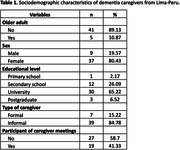# Effect of monthly training meetings about level of knowledge for dementia caregivers in Lima, Peru: preliminary results

**DOI:** 10.1002/alz.086229

**Published:** 2025-01-09

**Authors:** Belen Custodio, Diego Chambergo‐Michilot, Rosa Montesinos, Katherine Agüero, Graciet Verástegui, Nilton Custodio

**Affiliations:** ^1^ Instituto Peruano de Neurociencias, Lima, Lima Peru; ^2^ Unidad de diagnóstico de deterioro cognitivo y prevención de demencia, Instituto Peruano de Neurociencias, Lima, Perú, Lima, Lima Peru

## Abstract

**Background:**

Dementia is considered a public health problem due to the exponential increase in cases in recent years, as it not only has an impact on health services, but also affects the social and economic level, especially in low and middle income countries. Evidence has shown that a trained caregiver improves the patient’s quality of life, reduces behavioural symptoms and decreases the likelihood of the caregiver developing burnout. Currently scarce research has reported on the level of knowledge of dementia, specifically targeting caregivers in LMIC, that’s why we aimed to measure the level of knowledge of caregivers attending to monthly training compared to untrained caregivers.

**Method:**

This is a cross‐sectional analysis, comparing the level of knowledge between trained and untrained dementia caregivers in Lima, Peru. The training program (Lonchecito para cuidadores) consists of 10 virtual meetings per year, led by a healthcare professional, where the main topics are: types of dementia, management of behavioural symptoms, application of non‐pharmacological measures and caregiver well being. The instrument used was the Dementia Knowledge Assessment Scale (DKAS‐Spanish version), a validated scale with 25 true or false statements. A Student’s T test was performed to compare the mean scores of DKAS‐S between the trained versus untrained caregivers.

**Result:**

To date, 46 caregivers have participated in the study, representing 45% of the total sample. 80% of caregivers are women and 85% are informal caregivers. (Table 1) The preliminary results show a difference of two points on DKAS‐S between trained (mean score 16.22 ± 2.29) compared with untrained caregivers (mean score 18.21 ± 3.15). (Figure 1) A relevant finding is that 65% or more of the caregivers answered incorrectly questions 13, 15 and 19, which are related to onset symptoms of dementia, care in advanced stages and management of behavioural symptoms, respectively.

**Conclusion:**

The training program (Lonchecito para cuidadores) provides an increase in dementia knowledge. Training makes a difference between the two groups of caregivers, a reinforcement or different approach should be considered for the topics where both groups had the highest number of incorrect answers.